# Supporting Occupational Therapists in Predischarge Home Visit Decision-Making: Development and Evaluation of a Decision-Making Support Tool

**DOI:** 10.1155/oti/2296340

**Published:** 2025-02-26

**Authors:** Tammy Aplin, Maureen Godfrey, Liana De Michele, Amelia Hoffman, Claire Palmer, Christine McCormack, Courtney Halin, Michelle King, Jacqueline Nix, Amy Eldridge, Sally Eames

**Affiliations:** ^1^School of Health and Rehabilitation Sciences, The University of Queensland, St Lucia, Queensland, Australia; ^2^Allied Health Research Collaborative, The Prince Charles Hospital, Metro North Health, Chermside, Queensland, Australia; ^3^Occupational Therapy Department, The Prince Charles Hospital, Metro North Health, Chermside, Queensland, Australia; ^4^Occupational Therapy Department, Royal Brisbane and Women's Hospital, Metro North Health, Herston, Queensland, Australia; ^5^Brighton Health Campus, Metro North Health, Brighton, Queensland, Australia; ^6^Occupational Therapy Department, Redcliffe Hospital, Metro North Health, Redcliffe, Queensland, Australia; ^7^Metro North Health, Herston, Queensland, Australia; ^8^Community and Oral Health, Metro North Health, Brighton, Queensland, Australia

**Keywords:** assistive technology, clinical reasoning, home modification, home visit, occupational therapy

## Abstract

**Introduction:** With little evidence to guide practice, the decision to conduct a predischarge home visit poses challenges for occupational therapists. This study is aimed at evaluating the impact of a newly developed support tool for home visit decision-making on therapists' confidence, ease, and accuracy in decision-making and stakeholders' satisfaction in the communication of the decision and examining its clinical utility and reliability.

**Method:** The predischarge home visit decision-making support tool was trialed by occupational therapists from five facilities across an Australian metropolitan health service. Using a pre–post study design, therapists completed a purposefully developed questionnaire on decision-making practice including ease and confidence in decision-making. Accuracy of decision-making was also evaluated using case studies. Clinical utility and reliability data was also gathered.

**Results:** While therapists' confidence and ease in decision-making did not change with use of the tool, accuracy in decision-making improved regardless of therapist's experience in home visiting. The interrater reliability of the tool was moderate, with a Fleiss' kappa value of 0.51. Good internal consistency was demonstrated with the removal of one item (*α* = 0.83). Clinical utility was supported with therapists rating the tool as timely and easy to use.

**Conclusion:** The developed tool offers therapists a reliable tool to support clinical practice, by providing guidance in clinically reasoning the decision to conduct a home visit.

## 1. Introduction

Predischarge home visits form part of daily practice in a range of hospital settings. However, their resource-intensive nature means the decision to conduct a home visit must be well reasoned. To support occupational therapists in making this decision in practice, a predischarge home visit decision-making support tool was developed. This study had two aims: (1) examining the developed tool's impact on home visit decision-making practice including therapists' confidence, ease, and accuracy in decision-making and consumer's and the multidisciplinary teams' satisfaction in the communication of the decision and (2) evaluating the tool's clinical utility and reliability.

Home visits are a complex intervention completed by occupational therapists across a variety of settings. In the hospital context, home visits facilitate discharge planning and can assist in determining if patients can return home [1, 2]. These predischarge home visits allow occupational therapists to assess the person in their meaningful home environment, from which they can provide targeted education and advice to promote safety and participation in activities at home, including equipment and home adaptations [1–4]. Predischarge home visits offer numerous potential benefits, with research indicating they can improve participation in activities of daily living [5], reduce hospital admissions [2] and falls [6], and improve adherence to recommendations [7]. They have also been found to alleviate the concerns of family members, identify issues for discharge that were previously not identified [4], and facilitate collaboration between the therapist and client in rehabilitation goals [8].

Predischarge home visits can be an important part of a patient's recovery and success in returning home. However, limited hospital resources and the time- and resource-intensive nature of home visits [9–12] suggest they should be conducted only when beneficial to the patient. A recent systematic review indicated the need to be judicial in the decision to conduct predischarge home visits finding inadequate evidence to support their routine completion [3]. Predischarge home visits however have been found to be beneficial when well targeted, with a study of 198 community-dwelling older adults presenting to emergency with falls finding that occupational therapy home visits significantly reduced falls [13].

The diversity of individuals as well the cost and time intensity of home visits makes the decision of whom to conduct a home visit with challenging. To ensure consistency of practice, Lockwood et al. [11] suggested the use of evidence-based criteria to guide decision-making. In line with this recommendation, Boronowski et al. [14] developed a screening tool for the community hospital context to support decision-making. The tool, named the Occupational Therapy Discharge Needs Screen (OTDNS), identifies clients with more complex discharge-planning needs to assist in deciding whether hospital or community therapists would be more appropriate to complete home visits. It contains seven items, under the two categories of functioning and disability (medical conditions, patient knowledge, mobility, activities of daily living) and contextual factors (social support, physical/environmental barriers, and perceived readiness for discharge by patient and family). The tool has been found to be reliable and sensitive enough to indicate a score, 7 or above, of when a home visit is indicated [14]. While the tool shows good evidence of use in practice in the context of its development, its generalizability to wider contexts is yet to be investigated.

While the OTDNS presents a promising tool for practice, work by the current studies' authors which explored decision-making for predischarge home visits in Australian hospital settings found that while therapists wanted a tool to support decision-making, particularly for novice therapists, they wanted flexibility in decision-making to allow their clinical judgment to be incorporated and that the decision should not be guided by a prescriptive or score-based criteria [8]. The research team therefore concluded that a decision-making support tool which incorporated similar categories to the OTDNS but followed a less prescriptive approach to home visit decision-making would support practice in their context [8]. This present study builds on this earlier work [8] to develop and evaluate a decision-making support tool to guide therapists in predischarge home visit decision-making in this hospital setting in Queensland, Australia. The study had two aims: (1) evaluating the developed tool's impact on decision-making practice, including therapists' confidence, ease, and accuracy in decision-making, along with consumer and multidisciplinary team member's satisfaction in the communication of the decision, and (2) examining the tools clinical utility and reliability.

## 2. Methodology

### 2.1. Design

The study was a pre–post study design evaluating the use of the developed predischarge home visit decision-making support tool on a range of outcomes related to therapist practice, including therapist's ease, confidence, and accuracy in decision-making. The reliability and clinical utility of the tool were also evaluated. Ethical approval was granted from the Hospital Human Research Ethics Committee in 2018—Approval Number HREC/18/QPCH/33.

### 2.2. Context

The project was conducted across five sites in a Queensland Health Service District in Australia. These sites included two tertiary hospitals, two regional hospitals, and one slow stream inpatient rehabilitation setting. The tool was developed by the research team after identification of this as a key strategy to improve home visiting practice [8]. The team included occupational therapists from all sites, including senior and novice therapists with a range of experience in home visiting practice.

### 2.3. Predischarge Home Visit Decision-Making Support Tool

The foundation for the tool was the Person, Environment, Occupation Model [15]. Over several meetings, the research team identified and categorized key criteria that influenced decision-making under person, environment, and occupation domains from literature and their professional expertise. Ongoing meetings with the research team resulted in 10 items across the three domains. This included six items for the person domain: medical history, motor function, cognition, sensation/perception, discharge goals, and behavior/psychological status; two items for the domain of the environment, the physical and social environment; and the third domain of occupation included two items, personal activities of daily living and instrumental activities of daily living (including community access and leisure activities). The tool was piloted with eight patients and feedback sought on their perceptions, along with senior occupational therapy staff, with minor changes made to wording as a result.

A traffic light matrix system, as can be seen in Supporting Information (available here), was utilized in the tool to assess the overall picture of whether a home visit is indicated. Green indicates a low or unlikely need, yellow indicates a moderate need, and red is high or very likely need for a predischarge home visit for each criterion. Once each criterion of the matrix has been completed, the overall distribution of the traffic lights is used to support or guide decision-making. An outcome that would indicate the need for predischarge home visit, for example, would be all or almost all red traffic lights. An outcome that would recommend/indicate a predischarge home visit may not be required, for example, would be all green traffic lights. The tool however was designed to support decision-making and as such does not provide a definitive yes/no answer as to whether a predischarge home visit is required. This was intentional, to provide a tool that was to be used as a guide, not a prescriptive tool, as a matrix of all green, except one yellow or red may still indicate the need for a home visit if this item is significant for that individual.

If a predischarge home visit is not recommended/indicated using the support tool, the tool prompts the therapist to consider alternative means of home-based assessment. These additional options are listed at the end of the support tool and include an access visit (home visit without the patient present to assess the home environment conducted by an occupational therapist [9]), an access visit conducted by an occupational therapy assistant or allied health assistant, or referral to another service provider. There is also an option to indicate whether the access visit is completed virtually or in person, acknowledging the increasing use of technology-supported care [16, 17].

### 2.4. Participants

Occupational therapists were recruited from the five sites of a Queensland Health Service District across all acute and subacute facilities. The therapists were recruited by site champions from the research team via email, phone, or face-to-face communication. All therapists from each site were eligible to participate, having experience in conducting home visits, and were therefore invited to participate.

### 2.5. Procedure

**Figure 1:**
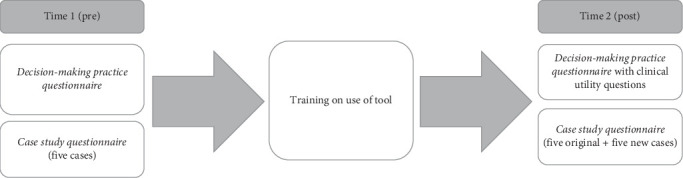
Outline of research procedure.

Therapists were invited to participate in the study which involved the completion of two questionnaires pre and post being trained in using the support tool as outlined in Figure 1. The first questionnaire was the *decision-making-practice questionnaire*. This questionnaire was completed by the therapist multiple times over the day, as they completed one for each client they worked with during a 1-day period. The second questionnaire was the *case study questionnaire* which was completed online at a separate time at the therapist's convenience. Given the questionnaires were completed at different timepoints, consent was sought separately. A participant information sheet and consent form for the *decision-making practice questionnaire* was provided by therapist site champions which was returned to the research team. For the *case study questionnaire*, participants were provided with an online link to the questionnaire which included participant information, consent, the case studies, and questionnaire.

#### 2.5.1. Time 1

Therapists completed the *decision-making practice questionnaire* for each client on the therapist's caseload list during a 1-day period without using the support tool. All participating therapists completed the questionnaires over the same day. This questionnaire asked therapists to make a home visit decision for each client in their caseload along with questions about the ease and confidence in their decision. It also involved therapists inviting the patients and a multidisciplinary team member to complete a single question item relating to that occupational therapist's communication regarding the decision to complete or not complete a home visit. The *case study questionnaire* was also completed at the therapist's convenience during this Time 1 period. The purpose of the case study questionnaire was to examine whether therapists were accurate in their decision-making using the tool. In this first time period, it involved therapists making a home visit decision for five purposefully developed case studies without using the support tool.

#### 2.5.2. Training

After Time 1's data collection, occupational therapists were provided with *training* on the development and use of the decision-making support tool. This involved a presentation from the site champions and an opportunity to ask questions and trial the use of the tool.

#### 2.5.3. Time 2

After this training, therapists were invited again to complete *the decision-making practice questionnaire*, with all clients over a 1-day period, this time using the support tool. They also completed the *case study questionnaire* again post the training and using the tool. This included the five original cases, as well as five new cases.

### 2.6. Data Collection Tools

#### 2.6.1. Decision-Making Practice Questionnaire

A purpose-developed questionnaire was utilized to measure therapist's ease and confidence in home visit decision-making and multidisciplinary team members' and consumers' perspective on communication of the decision made. The questionnaire included therapist demographic information including years of home visit experience, years of clinical experience, and gender. Patient demographic information for whom therapists completed the tool with was also recorded including their facility, ward (acute/subacute), age, gender, medical diagnosis, comorbidities, and mobility status. The questionnaire included questions on whether the decision was made to conduct a predischarge home visit and questions on ease and confidence in the decision using a 5-point Likert scale (1–5), for example, the ease scale ranged from “*very difficult*” to “*very easy*.” Time 2 questionnaire had additional questions on clinical utility. These included how much time using the tool added to practice, using a 5-point Likert scale from “*no time at all*” to “*a significant amount of time*,” if this time was valuable (yes/no), and how easy the tool was to use, on a 5-point Likert scale from “*not at all easy*” to “*very easy*” to use.

#### 2.6.2. Case Study Questionnaire

To evaluate the accuracy of decision-making, the case study questionnaire was developed. The questionnaire included basic demographic information including years of home visit experience, years of clinical experience, and gender. The questionnaire then presented five case studies where participants were requested to make the decision as to whether a predischarge home visit should be completed on not. The original five case studies were presented to the therapists for Time 2, when they were using the support tool along with an additional five new case studies. The case studies were developed by the research team to ensure a broad range of cases, including easy and difficult cases regarding home visit decision-making. To determine the “correct” response for each case, a consensus by a panel of three experienced therapists from the research team rated the case studies and where discrepancies occurred these were discussed until a consensus was reached.

### 2.7. Data Analysis

Demographic information along with consumer and multidisciplinary team (MDT) satisfaction with communication of the home visit decision was analyzed descriptively using Excel 2016. All inferential analysis was completed in SPSS 26.

#### 2.7.1. Ease and Confidence Analysis

Each therapist's responses to Likert scale data for ease and confidence in decision-making from the *decision-making practice questionnaire* were averaged across the number of clients they completed the questionnaire with, to have an average measure of ease and confidence score for each therapist. These average/mean scores allowed paired sample *t*-tests to be conducted to examine the differences between Time 1 and Time 2. Pearson's correlation was used to explore the relationship between therapist's level of experience and how easy and confident they were in making their decisions where an *r* value between 0.1 and 0.29 indicates a small relationship, *r* = 0.30–0.49 a medium relationship, and *r* = 0.5–1.0 a strong relationship [18].

#### 2.7.2. Accuracy Analysis

Paired sample *t*-tests were also used to examine change in the number of therapist's correct responses from Time 1 to Time 2 for the case studies. Independent sample *t*-tests were used to examine the influence of home visit experience on correct decisions, with a *p* value of ≤ 0.05 indicating statistical significance.

#### 2.7.3. Reliability and Clinical Utility Analysis

The *case study questionnaire* data from Time 2, when therapists used the support tool, was used for the interrater reliability analysis. The completed support tool responses from Time 2 decision-making practice questionnaire were used in the internal consistency analysis. Fleiss' kappa was used to explore interrater reliability as there were multiple raters. A Fleiss's kappa rating below 0.41 indicates poor to fair agreement, between 0.41 and 0.6 indicates moderate agreement, 0.61 and 0.8 good agreement, and 0.8–1.0 excellent agreement [19]. Internal consistency was examined using Cronbach's alpha, where ≥ 0.9 = excellent, 0.9–0.8 = good, 0.8–0.7 = acceptable, 0.7–0.6 = questionable, 0.6–0.5 = poor, and < 0.5 = unacceptable internal consistency [20].

## 3. Results

Across the five sites, 53 occupational therapists completed the *decision-making practice questionnaire* with 363 patients prior to using the tool at Time 1 and 32 therapists with 209 patients at Time 2. Items were not mandatory to complete on the *decision-making practice questionnaire* and as such some questions have fewer than 363 and 209 ratings at each time period. On average, therapists completed the *decision-making practice questionnaire* with 6.9 patients at Time 1 and 6.5 at Time 2. Of the 25 therapists who completed both Time 1 and Time 2 decision-making practice questionnaires, most were female (84%, *n* = 21) and worked with a subacute (48%, *n* = 12), acute (28%, *n* = 7), or both caseloads (24%, *n* = 6). For this group who completed both questionnaires, 44% (*n* = 11) were novice therapists, with 2 or less years' experience in home visiting and 56% (*n* = 14) were experienced therapists in home visiting practice. The *case study questionnaire* was completed by 28 therapists at Time 1 and Time 2, of which 50% (*n* = 14) were novice therapists and 86% (*n* = 24) were female.

### 3.1. Confidence and Ease in Decision-Making

Overall, therapists were confident in their decision to conduct a home visit both prior to and when using the decision-making support tool. Therapists reported feeling confident for 95% of their decisions (340/358 patient decisions) prior to using the tool and 93% (191/205 patient decisions) after the using the tool. Similarly, they reported finding the decision easy both prior to and when using the tool, with 91% of decisions (328/359 patient decisions) with their patients reported as easy prior to and 91% (185/204 patient decisions) after using the tool.

A paired sample *t*-test of the average confidence of the 25 therapists who completed Time 1 and Time 2 provides further evidence that confidence and ease in decision-making were high for therapists regardless of use of the tool. No significant difference was found between therapists' average in ease of decision-making across Time 1 (*M* = 4.50, SD = 0.54) and Time 2 (*M* = 4.57, SD = 0.47), *t*(25) = −0.614, *p* = 0.545. Similarly, there was no significant change in confidence in being able to justify their decision between Time 1 (*M* = 4.61, SD = 0.50) and Time 2 (*M* = 4.60, SD = 0.45), *t*(25) = 0.017, *p* = 0.986.

There was a moderate correlation between therapist's level of experience and how easy they found the decision for both time points. Pearson's correlation showed a medium relationship between therapist's experience level and their ease in decision-making for both Time 1 (*r* = 0.427, *n* = 25, *p* = 0.033) and Time 2 (*r* = 0.427, *n* = 25, *p* = 0.033), with therapists with more experience associated with therapists reporting the decision was easier to make. For Time 2 (using the tool), a significant relationship was found between therapists' level of experience and confidence (*r* = 0.460, *n* = 25, *p* = 0.021), where therapists with more experience were more likely to report more confidence in their decision; this was found to be trending for Time 1 as well; however, it was not significant (*r* = 0.373, *n* = 25, *p* = 0.064).

### 3.2. Accuracy in Decision-Making

Although the tool appeared to have little impact on therapist's confidence or their perception of ease in making the decision as to whether a home visit should be conducted, the accuracy of their decision appears to be influenced by using the tool. There was a significant increase in therapist's correct decisions between Time 1 and Time 2 when using the decision-making support tool to complete the case studies in the *case study questionnaire*. A paired sample *t*-test revealed a significant increase in correct decisions to conduct a home visit from Time 1 (*M* = 3.43, SD = 1.07) to Time 2 for the repeated cases (*M* = 3.93, SD = 0.60), *t*(27) = −2.32, *p* = 0.028, and the new cases (*M* = 4.36, SD = 0.87), *t*(27) = −3.77, *p* = 0.001. The independent sample *t*-test revealed no significant difference between novice and experienced therapists in correct decisions prior to or when using the tool for the repeated or new cases.

### 3.3. Consumer and MDT Satisfaction

**Figure 2:**
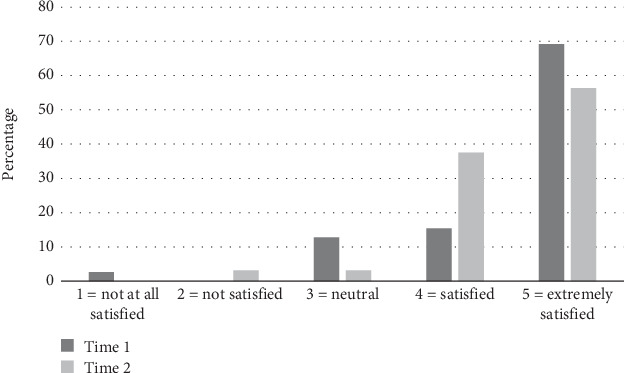
Consumer satisfaction with communication about need to conduct a home visit.

**Figure 3:**
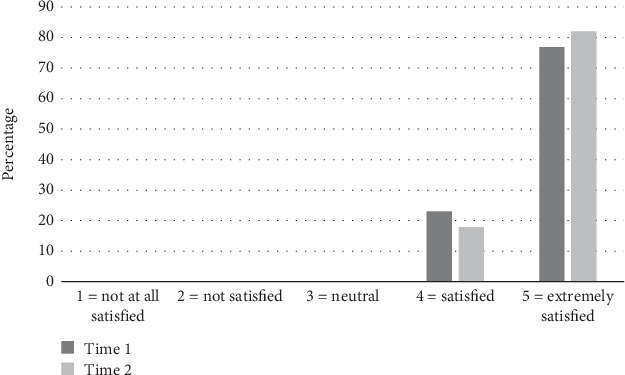
Multidisciplinary team members' satisfaction with communication about need to conduct a home visit.

Both consumers and the MDT team were satisfied with communication about the need to conduct a home visit both prior to and when using the tool. Only one patient prior to and one after using the tool were dissatisfied and no members of the MDT team reported being dissatisfied with communication at either time point, as demonstrated in Figures 2 and 3.

### 3.4. Clinical Utility and Reliability

Of the 32 therapists who completed the *decision-making practice questionnaire* when using the decision-making support tool (Time 2), 27 responded to items on the clinical utility of the tool. Of these therapists, 93% (*n* = 25) felt the tool took no time at all to a moderate amount of time to complete and 74% (*n* = 20) felt that the time spent to complete the tool was valuable. Most (67%, *n* = 18) found the tool easy to complete; however, some found it not as easy with 33% (*n* = 9) rating this as neutral.

The interrater reliability of the tool was found to be moderate, with a Fleiss' kappa value of 0.508. Cronbach's alpha on initial analysis for the tool was *α* = 0.2 indicating poor reliability. However, investigation of the item statistics during analysis revealed that removing the item “medical history” significantly improved the reliability of the tool. Discussion with the research team during this analysis stage supported removal of this item, as medical history can include many factors such comorbidities that do not significantly impact the patient's current status. It was discussed that those factors which are relevant which were assessed under the item “medical history” such as length of hospital admission would be captured by other items in the tool. Cronbach's alpha improved significantly with the removal of this item to *α* = 0.83 indicating good internal consistency. The updated version of the decision-making support tool after this study has this item removed.

## 4. Discussion

This study is aimed at evaluating the use of the newly developed *predischarge home visit decision-making support tool's* impact on therapist's confidence, ease, and accuracy in home visit decision-making, along with consumer and multidisciplinary team member's satisfaction in the communication of the decision. The tool was found to have little impact on confidence and ease in decision-making with therapists being confident with and without using the tool. However, accuracy in decision-making increased when using the tool, suggesting there may be benefit of using the tool in daily practice. Satisfaction with communication prior to and when using the tool did not change, with high satisfaction from consumers and the multidisciplinary team prior to the use of the tool and when using it. The findings of the secondary aim of the study to examine the tool's reliability and clinical utility provide initial evidence of the tool's reliability and that it is easy to use in practice.

Therapists were confident in their decisions with or without using the tool, and this is likely a result of their overall high levels of confidence in commonly making this decision. However, the finding that the tool increased accuracy in decision-making points to the complex nature of home visit decision-making and that the tool may enhance practice even for experienced and confident therapists. The enhanced accuracy using the tool may suggest that therapists on occasion can make quick or habituated decisions without considering a range of person, environment, and occupation factors for the individual. The tool therefore offers therapists and teams a valuable resource for identifying patients in need of home visits, along with consistency in the decision-making process. The tool provides a resource for therapists to ensure they consider the wide array of factors which influence an individual's need for a home visit as it prompts clinical reasoning for each factor. The use of the tool in practice may also enhance person-centered practice by drawing the therapist's attention to patient factors, rather than focusing on the need for discharge or prioritizing organizational and resource factors, such as time, access to transport, and clinical setting expectations, which have been shown in the literature to influence health professional's home visit decision-making and patient care [4, 8, 11, 21]. While there was no change in MDT communication satisfaction in the findings, the tool may assist clinicians to advocate within their multidisciplinary teams for a home visit to be completed before discharge and reminds both treating clinicians and the team of alternative options, such as telehealth home visits.

The benefit of using the tool in practice was demonstrated by accuracy in decision-making improving for both novice and expert therapists. This result was expected for novice therapists, who benefit from structed processes to support their clinical reasoning [22], and indicated that a tool to support home modification decision-making would be valuable [8]. The increased accuracy for expert therapists in contrast was somewhat unexcepted. There are however a number of therapist factors unrelated to level of experience, such as motivation to work and interest in their specialization, along with environmental factors such as limited facilities and inadequate patient to staff ratios, which have been found to influence the clinical reasoning of occupational therapists [23]. This may explain the current study's findings that while expert therapists are confident in their decisions, given the range of other factors that may be influencing their home visit decision-making, the tool is valuable in their practice. This finding suggests that tools to support complex clinical reasoning decisions are valuable for all therapists to ensure decisions are well considered and that external factors such as time pressures do not negatively influence decisions.

The decision to remove the medical history item from the tool was decided by the research team as it incorporates a number of aspects of the person which may not be related to their current health status and ability to return home. This decision aligns with Lockwood et al.'s [11] study which investigated factors associated with home visit completion for patients following hip fracture. Comorbidities were not found to be related which is reflective of the medical history item in the tool [11]. While this information may not be essential in making a predischarge home visit decision, it is important for comprehensive clinical communication in the transition from hospital to home care. It is also important to consumers, who have reported that it is important for treating clinicians to have an understanding of their medical history during this transition period [24].

### 4.1. Limitations and Future Research

The *predischarge home visit decision-making support tool* was developed from an identified need within the clinical setting. Developed across a large health service, which includes acute, subacute, and long-term rehabilitation settings, the tool strength lies in its development close to its users. However, the process of development would have been enhanced using an evidence-based method such as the Delphi method. The tool was also trialed with adequate number of therapists with support for interrater reliability. However, limitations in the study design should be acknowledged. A critical weakness of the study is that the accuracy analysis was based on clinical consensus of “correct” decisions from only three expert therapists, and caution should therefore be applied to the validity of the results. Further, given therapists were aware of the tool's development from an identified clinical need in their practice area, they may have been biased in their responses regarding clinical utility and thus future research should seek to examine clinical utility and reliability across wider hospital settings. It may also be useful to examine the usefulness of the tool to support the referral process between hospital and community services along with its usefulness in community settings for prioritization of home visits.

Future research should also seek to explore the impact of the tool's use on practice outside of therapist's experience, for example, it will be important to evaluate the impact of the tool's use on client outcomes and resource utilization. Ongoing use of the tool in clinical settings may also provide valuable information to inform home visiting practice, to understand who is receiving and most benefiting from home visits. The results indicated that some therapists found the tool not easy to use, suggesting that training in the tool is necessary to support its clinical implementation. The training which was provided to participants in this research study had limited time allocated for application. In response to these findings and therapist feedback, an online training module with interactive clinical scenarios has been developed for future use. The use of satisfaction measures for measuring consumer and multidisciplinary team member perspectives on the tool may have limited the study, with overall high ratings of satisfaction both with and without use of the tool. Future research would be warranted to examine the tool's impact on multidisciplinary team members' understanding and acceptance of occupational therapists' recommendations for home visits using more robust measures.

## 5. Conclusion

This study finds preliminary evidence of the valuable role the newly developed *predischarge home visit decision-making support tool* may have in supporting occupational therapists to clinically reason when to conduct a predischarge home visit in hospital settings. Further research is now needed exploring consumer outcomes and the tool's impact on resource utilization to support its use. The tool however provides a promising resource for hospital settings for prioritizing the time- and resource-intensive practice of home visiting. The study found that while the tool does not enhance therapists' confidence or ease in decision-making, likely because therapists had prominent levels of confidence previously, their accuracy in decision-making improves using the tool regardless of experience in home visiting practice. The tool was found to have acceptable interrater reliability and internal consistency, along with support for clinical utility with results indicating it is easy to use in practice.

## Data Availability

Data for this research may be available by request from the authorship team.

## Supporting Information

Supporting Information Additional supporting information can be found online in the Supporting Information section. The supporting information contains the research version of the predischarge home visit decision-making support tool. The final version does not include the medical history item.

## References

[1] Chibnall C. (2011). Are home assessments beneficial in discharge planning following a stroke? A viewpoint. *Australian Occupational Therapy Journal*.

[2] Lockwood K. J., Taylor N. G., Harding K. E. (2015). Pre-discharge home assessment visits in assisting patientsâ€™ return to community living: a systematic review and meta-analysis. *Journal of Rehabilitation Medicine*.

[3] Fukumoto M., Watanabe T., Yasufuku Y., Furudate K., Momosaki R. (2019). Home visits by occupational therapists in acute hospital care: a systematic review. *International Journal of Rehabilitation Research*.

[4] Whitehead P., Fellows K., Sprigg N., Walker M., Drummond A. (2014). Who should have a pre-discharge home assessment visit after a stroke? A qualitative study of occupational therapists’ views. *British Journal of Occupational Therapy*.

[5] Lannin N. A., Clemson L., McCluskey A., Lin C.-W. C., Cameron I. D., Barras S. (2007). Feasibility and results of a randomised pilot-study of pre-discharge occupational therapy home visits. *BMC Health Services Research*.

[6] Lockwood K. J., Harding K. E., Boyd J. N., Taylor N. F. (2019). Predischarge home visits after hip fracture: a randomized controlled trial. *Clinical Rehabilitation*.

[7] Lockwood K., Harding K., Boyd J., Taylor N. F. (2020). Home visits by occupational therapists improve adherence to recommendations: process evaluation of a randomised controlled trial. *Australian Occupational Therapy Journal*.

[8] Godfrey M., Cornwell P., Eames S., Hodson T., Thomas T., Gillen A. (2019). Pre-discharge home visits: a qualitative exploration of the experience of occupational therapists and multidisciplinary stakeholders. *Australian Occupational Therapy Journal*.

[9] Atwal A., Spiliotopoulou G., Stradden J. (2014). Factors influencing occupational therapy home visit practice: a qualitative study. *Scandinavian Journal of Occupational Therapy*.

[10] Clemson L., Lannin N. A., Wales K. (2016). Occupational therapy predischarge home visits in acute hospital care: a randomized trial. *Journal of American Geriatrics Society*.

[11] Lockwood K. J., Taylor N. F., Boyd J. N., Harding K. E. (2017). Pre-discharge home visits by occupational therapists completed for patients following hip fracture. *Australian Occupational Therapy Journal*.

[12] Sampson C., James M., Whitehead P., Drummond A. (2014). An introduction to economic evaluation in occupational therapy: cost-effectiveness of pre-discharge home visits after stroke (HOVIS). *British Journal of Occupational Therapy*.

[13] Chu M. M., Fong K. N., Lit A. C. (2017). An occupational therapy fall reduction home visit program for community-dwelling older adults in Hong Kong after an emergency department visit for a fall. *Journal of the American Geriatrics Society*.

[14] Boronowski L. E., Shorter C. M., Miller W. C. (2012). Measurement properties of the occupational therapy discharge needs screen. *Canadian Journal of Occupational Therapy*.

[15] Law M., Cooper B., Strong S., Stewart D., Rigby P., Letts L. (1996). The person-environment-occupation model: a transactive approach to occupational performance. *Canadian Journal of Occupational Therapy*.

[16] Nix J., Comans T. (2017). Home Quick–occupational therapy home visits using mHealth, to facilitate discharge from acute admission back to the community. *International Journal of Telerehabilitation*.

[17] Read J., Jones N., Fegan C. (2020). Remote home visit: exploring the feasibility, acceptability and potential benefits of using digital technology to undertake occupational therapy home assessments. *British Journal of Occupational Therapy*.

[18] Pallant J. (2016). *SPSS Survival Manual: A Step By Step Guide to Data Analysis Using SPSS Program*.

[19] Altman D. G. (1999). *Practical Statistics for Medical Research*.

[20] George D., Mallery P. (2005). *SPSS for Windows Step by Step: A Simple Guide and Reference 12.0 Update*.

[21] Drummond A., Whitehead P., Fellows K. (2012). Occupational therapy pre-discharge home visits for patients with a stroke: what is national practice?. *British Journal of Occupational Therapy*.

[22] Unsworth C. A. (2001). The clinical reasoning of novice and expert occupational therapists. *Scandinavian Journal of Occupational Therapy*.

[23] Shafaroodi N., Kamali M., Parvizy S., Mehraban A. H., O'Toole G. (2014). Factors affecting clinical reasoning of occupational therapists: a qualitative study. *Medical journal of the Islamic Republic of Iran*.

[24] Brown S., Craddock D., Greenyer C. H. (2012). Medical patients’ experiences of inreach occupational therapy: continuity between hospital and home. *British Journal of Occupational Therapy*.

[25] Aplin T., Godfrey M., De Michele L. (2021). Supporting occupational therapists in pre-discharge home visit decision-making: Development and evaluation of a decision-making support tool. Occupational Therapy Australia, 29th National Conference and Exhibition, “Inspired Insights for Brighter Futures”, 23–25 June 2021, Virtual Conference and Engagement Hubs. *Australian Occupational Therapy Journal*.

