# Supplementary material for: Supporting Occupational Therapists in Predischarge Home Visit Decision-Making: Development and Evaluation of a Decision-Making Support Tool
Source: Occup Ther Int. 2025 Feb 26;2025:2296340. doi: 10.1155/oti/2296340 (PMC11985242; doi:10.1155/oti/2296340)
Supplement: Supporting Information — Additional supporting information can be found online in the Supporting Information section. The supporting information contains the research version of the predischarge home visit decision-making support tool. The final version does not include the medical history item. [file 2296340.f1.doc]

**Pre-discharge Home Visit Decision Making Support Tool**

| **Clinical Factors** | **Low or unlikely need for Pre-discharge Home Visit** | **Moderate need for Pre-discharge Home Visit** | **High or very likely need for Pre-discharge Home Visit** |
| --- | --- | --- | --- |
| **PERSON DOMAIN** | | | |
| MEDICAL HISTORY | Negligible concerns regarding medical history (i.e. medically stable and/ or approximating preadmission/*baseline* status) | Moderate concerns with medical history (e.g. multiple comorbidities, long hospital admission) | Significant medical history (e.g. multiple comorbidities, multiple hospital admissions, progressive condition, significant change in medical status) |
| MOTOR FUNCTION  (Mobility, falls risk, UL/LL function) | Negligible concerns regarding motor function (e.g. negligible falls history/risk, return to preadmission mobility status) | Moderate motor concern (e.g. low to moderate falls history/risk, reduced endurance/balance, new mobility aid) | Significant motor concern (e.g. high falls history/risk, poor safety, significant change in mobility status, requiring supervision/physical assistance from carer) |
| COGNITION  (Orientation, attention, memory, insight, problem-solving, abstract reasoning) | Negligible concern regarding cognition (e.g. return to preadmission status) | Moderate cognitive concern (e.g. difference between observations in hospital environment versus reported behaviour at home) | Significant cognitive concern (e.g. family/carer reporting concerns, diagnosis of cognitive impairment) |
| SENSATION /PERCEPTION  (Vision, hearing, sensation, smell, new visual extinction/ inattention, hemianopia, nystagmus, neglect) | Negligible concerns regarding sensory/ perceptual function (e.g. return to preadmission status) | Moderate concerns regarding sensory/ perceptual function | Significant concern regarding sensory/ perceptual function (e.g. significant change in sensory function) |
| DISCHARGE GOALS | Realistic discharge goals | Client/carer unrealistic about some discharge goals; some level of discrepancy regarding discharge goals and expectations. | Client/ carer unrealistic about discharge goals/ destination |
| BEHAVIOURAL / PSYCHOLOGOICAL STATUS | Negligible concerns regarding behavioural / psychological status | Moderate concerns regarding behavioural / psychological status | Significant concerns regarding behavioural / psychological status |
| **ENVIRONMENT DOMAIN** | | | |
| PHYSICAL ENVIRONMENT  (Home set up, environmental hazards, equipment and modifications) | Negligible concerns regarding discharge environment (e.g. well set up and appropriate to client’s functional status) | Moderate concerns regarding discharge environment (e.g. new equipment or inappropriate equipment in place) | Significant concerns regarding discharge environment (e.g. inappropriate or inadequate modifications/ equipment/ access, need to trial new equipment (e.g. MASS), concerns raised by community services/ carer; inappropriate environment for carer/services) |
| SOCIAL ENVIRONMENT  (Carer burden/health, emotional environments, services, family /friends network) | Negligible concerns regarding family/support on d/c raised by client/carer/MDT (e.g. adequate social supports) | Moderate concerns regarding family/support on d/c raised by client/carer/MDT (e.g. carer able to provide intermittent assistance due to employment, own medical status) | Significant concerns regarding family/support on d/c raised by client/carer/MDT (e.g. carer cognitively compromised, no supports, elder abuse suspected, food safety issues) |
| **OCCUPATION DOMAIN** | | | |
| PADL - Occupational performance | Negligible concerns regarding  PADL completion (e.g. safe, independent; appropriate carer support or services) | Moderate concerns regarding safe completion of PADL (e.g. refusing assessment; refuses assistance) | Significant concerns regarding completion of PADL (e.g. poor standard of hygiene impacting on health; full assistance; significant change in PADL status, requiring supervision/physical assistance from carer) |
| IADL - Occupational Performance  (Including community access, leisure activities) | Negligible concerns regarding  IADL completion (e.g. safe, independent or appropriate carer support or services) | Moderate concerns regarding  IADL completion (e.g. refusing support/services) | Significant concerns regarding  IADL completion (e.g. malnourished, medication safety, no carer supports or services) |

| **Decision** | | | |
| --- | --- | --- | --- |
| Pre-discharge Home Visit (HV) recommended (please circle) | Yes | No | If **NO**, is an alternative method of Home Assessment recommended? YES/NO (circle)   1. Access Visit by OT (in home or virtual) 2. Access Visit by OTA / AHA (in home or virtual) 3. Other Service Provider |
